# Kinetic Modeling of ABCG2 Transporter Heterogeneity: A Quantitative, Single-Cell Analysis of the Side Population Assay

**DOI:** 10.1371/journal.pcbi.1005188

**Published:** 2016-11-16

**Authors:** Adam F. Prasanphanich, Douglas E. White, Margaret A. Gran, Melissa L. Kemp

**Affiliations:** The Wallace H. Coulter Department of Biomedical Engineering Georgia Institute of Technology and Emory University, Atlanta, Georgia, United States of America; Johns Hopkins University, UNITED STATES

## Abstract

The side population (SP) assay, a technique used in cancer and stem cell research, assesses the activity of ABC transporters on Hoechst staining in the presence and absence of transporter inhibition, identifying SP and non-SP cell (NSP) subpopulations by differential staining intensity. The interpretation of the assay is complicated because the transporter-mediated mechanisms fail to account for cell-to-cell variability within a population or adequately control the direct role of transporter activity on staining intensity. We hypothesized that differences in dye kinetics at the single-cell level, such as ABCG2 transporter-mediated efflux and DNA binding, are responsible for the differential cell staining that demarcates SP/NSP identity. We report changes in A549 phenotype during time in culture and with TGFβ treatment that correlate with SP size. Clonal expansion of individually sorted cells re-established both SP and NSPs, indicating that SP membership is dynamic. To assess the validity of a purely kinetics-based interpretation of SP/NSP identity, we developed a computational approach that simulated cell staining within a heterogeneous cell population; this exercise allowed for the direct inference of the role of transporter activity and inhibition on cell staining. Our simulated SP assay yielded appropriate SP responses for kinetic scenarios in which high transporter activity existed in a portion of the cells and little differential staining occurred in the majority of the population. With our approach for single-cell analysis, we observed SP and NSP cells at both ends of a transporter activity continuum, demonstrating that features of transporter activity as well as DNA content are determinants of SP/NSP identity.

## Author Summary

A common method of evaluating stemness among pluripotent cells or cancer cells is the side population assay, a flow cytometry technique which identifies a subgroup of cells that exhibit differences in dye fluorescence upon blocking of a membrane transporter. A technical limitation of this assay is that it relies on two independent experimental conditions, with and without a transporter inhibitor, preventing evaluation of single cell characteristics that generate population-level shifts in fluorescence. Here, the computational implementation of various forms of cellular heterogeneity allows for ensemble single-cell simulations to be performed in order to assess the underlying properties that give rise to the population-level behavior. We simulated staining in 10,000 kinetic ensembles consisting of 1,000-cell populations with and without inhibitor to determine which cells respond in the assay. We quantitatively establish that a small, responsive subgroup of cells with nonlinear activities associated with transporter number are most likely to recapitulate observed behavior in the side population assay; however, a continuum of phenotypes at different stages of the cell cycle and with a range transporter expression levels will shift fluorescence. We present a new perspective on the phenotype of SP cells at the single-cell level that is determined by biological and experimental kinetic processes, and is not equivalent to a cancer stem cell phenotype.

## Introduction

The side population (SP) assay is used to identify stem cells by flow cytometry through the characteristic of enhanced dye efflux mediated via ATP-binding cassette (ABC) transporters [1]. The SP was first identified by Goodell et al. as hematopoietic stem cells in samples of murine bone marrow aspirate [2]. The role of the SP has since expanded to serve as a means to identify stem cell populations based, primarily, on ABCG2 activity [3], though additional ABC transporters, such as P-glycoprotein/ABCB1, can also mediate formation of a SP [4]. ABCG2, also known as breast cancer resistance protein (BRCP), can mediate multidrug resistance (MDR) in breast [5–9] and other cell lines [10–14]. The SP has been implicated in numerous cancers as a harbinger of MDR-mediated chemoresistance [15–18] and cancer stem cells (CSCs) [19–22] in *in vitro* cancer cell lines; thus the presence of a SP is understood as an undesirable indicator.

**Fig 1:**
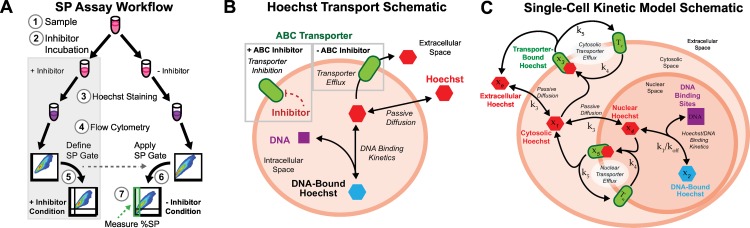
Hoechst Staining Overview and SP Assay Conceptual Model. (A) Experimental workflow of sample preparation and processing in a SP assay. Each sample (1) is split into two conditions, with (+inhibitor) and without (-inhibitor) the ABC transporter inhibitor (2). Both conditions are then stained with Hoechst 33342 (3) and the resultant fluorescence measured via flow cytometry (4). The +inhibitor condition is used to define a gate for the non-side population (NSP) region (5), which is then applied to the -inhibitor condition (6) to identify the SP region (green box), which is measured as percent of the parent population (7). (B) Schematic of the Hoechst transport processes presumed to underlie the SP assay. Hoechst 33342 passively diffuses into the cell, where it is transported out of the cell via transporter (-inhibitor condition) or binds to DNA. For the +inhibitor condition, a small molecule compound inhibits the transporter, preventing transporter-mediated efflux. (C) Hoechst staining dynamics were simulated at the single-cell level with each cell represented by a set of ODEs governed by mass-action kinetics in a well-mixed three-compartment system. The species, compartments, and reactions are depicted. Each cell differs from the rest of the population in terms of volumes, surface areas, transporter properties, and DNA content. Within a given population, all the cells share a common set of kinetic parameters (k).

SPs are identified by splitting samples into conditions with and without an ABC transporter inhibitor followed by Hoechst staining, which enables population-level comparison of differences in cell staining due to ABC transporter activity between the two conditions (Fig 1A). Blocking of transporter mediated Hoechst efflux by the inhibitor serves as a basis for comparison of cell staining in the condition without the transporter inhibitor (Fig 1B). When comparing the two conditions, SP cells are observed as a population with decreased staining in the lower left of the Hoechst Red and Blue staining plot (Fig 1A).

The basis of differential staining is thought to be driven by impaired dye efflux in the presence of ABC transporter inhibitor, with SP cells exhibiting high-ABC transporter activity and decreased staining compared to low-ABC transporter-activity NSP cells with uninhibited transporter activity [3,23,24]. Although the kinetic, ABC transporter-mediated mechanism is universally accepted as the basis for differential staining of cell populations in the +inhibitor and -inhibitor conditions of the SP assay, differences in cell staining due to transporter activity have not been demonstrated from a kinetic perspective nor at the single-cell level. This gap in knowledge persists due to a technical limitation that precludes an individual cell from Hoechst staining in both +inhibitor and -inhibitor conditions, thereby preventing any measurement of shifts of individual cells within the population distribution. Therefore, it is unclear how the heterogeneity of ABC transporter activity within a population influences staining characteristics in the +inhibitor and -inhibitor conditions of the SP assay and unclear how the heterogeneity in transporter activity is reflected in individual cells of the SP and NSP. We hypothesize that specific distributions of ABC transporter activity in a population, representing the heterogeneity of transporter activity at the single-cell level, will exhibit differential cell staining as is observed in the SP assay.

In this study, we employ experimental and computational approaches to demonstrate the kinetic nature of the SP assay and the dynamic nature of the SP/NSP phenotype. Experimentally, we investigated SP formation in the A549 lung carcinoma cell line with Hoechst 33342 staining and inhibition of ABCG2 with the inhibitor Fumitremorgin C (FTC) [25,26]. We present a novel computational approach for simulating heterogeneity in transport kinetics, using mass-action kinetic reactions inspired by the conceptual model of Hoechst staining the SP (Fig 1B and 1C), at the single-cell level across a population. The population-level model is used to demonstrate the validity of the transporter-mediated kinetic interpretation of the SP assay. The approach enables *in silico* staining of an identical cell population in both inhibitor-free (–FTC) and inhibitor-containing (+FTC) conditions with subsequent single-cell analysis of the role of transporter variability on cell staining. In this manner, we investigated the role of heterogeneity in transporter expression, activity, and kinetics at the single-cell level on the formation of the side population.

## Results

### SP Phenotype is Dynamic & Correlates with ABCG2 Expression

**Fig 2:**
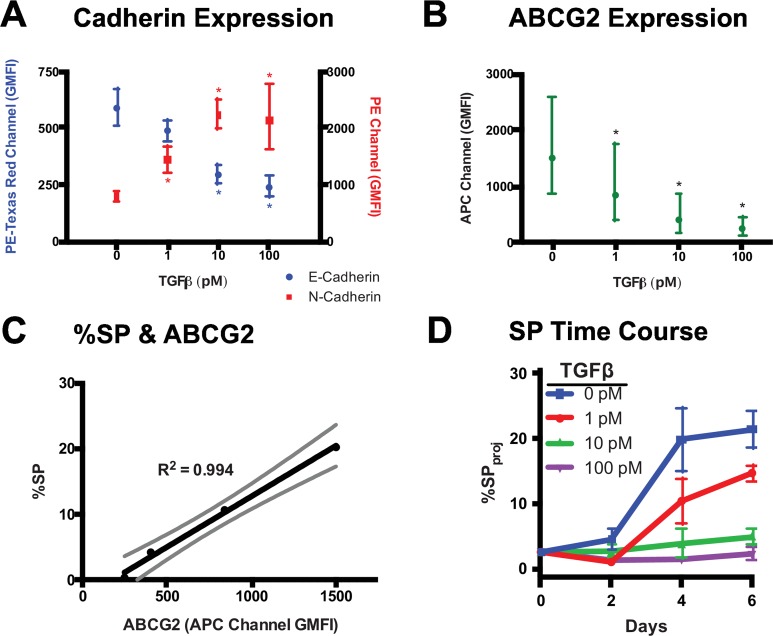
Surface Marker Staining & SP Size Following TGFβ-Mediated EMT. Surface marker expression measured via flow cytometry after staining with anti-PE-CF594/E-Cadherin, PE/N-Cadherin (A), and APC/ABCG2 (B) antibodies on live A549 cells following 4-day treatment with 0, 1, 10, and 100 pM TGFβ. Surface marker staining data were obtained from 3-color staining, with compensation, from 3 biological replicates. Values are plotted as the geometric mean ± 95% confidence interval of the sample geometric mean fluorescence intensities (GMFI). A significant difference from the 0 pM TGFβ condition was determined with a two-way ANOVA, p<0.05, and are indicated by asterisks (*). (C) Mean %SP versus ABCG2 expression for the Day 4 condition from the SP time course experiment, along with a best-fit line from a linear regression, R^2^ shown, and 95% confidence interval. (D) SP size measured by projection gating with increasing time in culture (up to 6 days) and TGFβ. Performed in triplicate and plotted as mean ± SEM.

A549 lung carcinoma cells were expanded in culture for 4 days prior to measurement of the SP by the Hoechst staining assay (Fig 1A). We observed an initial 18% SP in A549 cells (S1A Fig), which was eliminated by treatment with TGFβ (S1B and S1C Fig). TGFβ treatment for 4 days resulted in epithelial-mesenchymal transition (EMT), indicated by down-regulation of E-cadherin and up-regulation of N-cadherin (Fig 2A and S2 Fig), as well as down-regulation of ABCG2 (Fig 2B and S2 Fig). SP percentage was correlated with ABCG2 expression (Fig 2C).

We developed an algorithmic approach for objective quantification of SP size using a Hoechst staining threshold to define the gate that delineates SP and NSP regions (S3 Fig). Although suggestions have been made for standardizing the reporting of SP assay results [1], gating protocols vary considerable in the literature. Here, we defined Hoechst staining intensity as the x-y projection of z-score transformed Hoechst Red and Blue raw signals with a threshold set at the 1^st^ percentile in the +FTC condition. This projection gating approach demonstrated a strong correlation (R^2^ = 0.98, S4B Fig) between manual and automated algorithmic measurement of percentage SP.

To further elucidate the role of TGFβ on SP size, we made serial measurements of SP size using our projection gating method. A549 cells were expanded in culture for 4 days without TGFβ treatment and then passaged. On the subsequent day (Day 0), treatment with 0, 1, 10, and 100 pM TGFβ was started and SP size was measured at 2 day intervals (S4A Fig). On the day of passage, Day 4, the SP constituted 20% of the cell population; however, following cell passage, the SP consisted of 2% of the population (Fig 2D). In the following days, SP size increased and plateaued near 20% in the control condition. With increasing amounts of TGFβ exposure, the repopulation of the SP attenuated in a dose-dependent manner (Fig 2D).

We derived clonal populations of A549 cells by expanding individual low- and high-ABCG2 expressing cells to determine whether subclones within the A549 cell line define either the SP or NSP subpopulations. Cells were labeled with anti-ABCG2 antibody and 96 high-ABCG2 expressing and 96 low-ABCG2 expressing cells were sorted into individual wells of a 96 well–plate (S5A Fig). Most cells failed to form colonies; however, following expansion of individual cells for 30 days, all surviving cells demonstrated both SP and NSP cells (S5B Fig).

Collectively, these results indicate that the SP within the A549 cell line is dynamic with respect to cell phenotype (Fig 2A), cyclically variable in culture (Fig 2D), and is not genetically distinct from the NSP (S5 Fig).

### SP & NSP Are Not Distinct Subpopulations

The study of side populations has been limited by the approach to define the SP, which relied on manually defined gates to define a boundary between SP and NSP regions within the Hoechst Red and Hoechst Blue plot. In the development of our automated projection gating approach to measure SP size, we closely examined numerous aspects associated with SP data. A key observation was that the SP and NSP are not clearly separated within the–FTC condition. We observed a continuous staining distribution in both Hoechst Red and Blue channels of the–FTC condition, which is redistributed towards lower signal intensities compared to the +FTC condition (S6A and S6B Fig). The decrease of mean Hoechst signal intensity in the–FTC condition, compared to the +FTC condition, correlated with increased SP size (S6C Fig).

**Fig 3:**
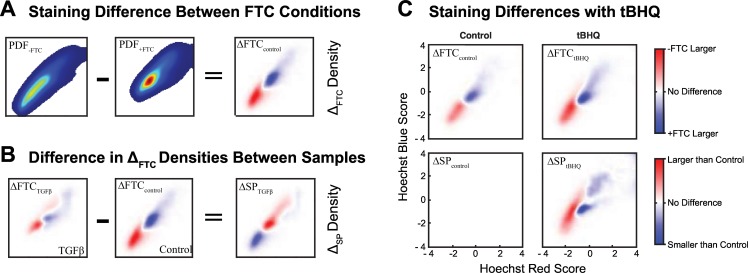
Visualizing Differences in Distributions with ΔFTC & ΔSP Plots. Differences in Hoechst Score PDFs are used to compare differences between +FTC and -FTC conditions as well as between samples. (A) The difference between PDF_-FTC_ and PDF_+FTC_ for the control sample is represented by the ΔFTC_control_ plot. (B) Differences between ΔFTC_control_ and ΔFTC_TGFβ_ are represented in the ΔSP_TGFβ_ plot. (C) Differences in PDF density between PDF_-FTC_ and PDF_+FTC_ are displayed as ΔFTC distribution plots for control and tBHQ-treated conditions plotted against Hoechst Red and Blue Scores. Differences in ΔFTC distributions from the control sample ΔFTC distribution are displayed as ΔSP distributions for the control and tBHQ-treated samples. ΔFTC and ΔSP distributions are averaged from three replicates.

The intensity of both Hoechst Red and Blue staining is greatly reduced in the conventional binary assignment of cells into SP or NSP subpopulations. We sought to develop analytical methods to enable characterization of staining data from the SP assay with greater preservation of staining information. We processed the Hoechst Red and Blue flow cytometry signals into 2-dimensional population density functions (PDF) for both the +FTC (PDF_+FTC_) and–FTC (PDF_-FTC_) conditions, which converted raw data into normalized data sets (S3 Fig). This allowed us to directly compare spatial differences in staining intensity in the +FTC and–FTC conditions by subtracting the PDF_+FTC_ from the PDF_-FTC_ to define the ΔFTC density for a given sample (Fig 3A). Conversion of the raw data into PDFs was critical because it allowed for direct comparison of flow cytometry samples of un-equal event counts. An increased cell population density in the–FTC condition compared to the +FTC condition is visualized as a red signal whereas blue indicates decreased population density. By converting raw data into PDFs and thereby normalizing, we able to make quantitative comparisons in spatial staining intensities between +FTC and–FTC conditions of a particular sample. Furthermore, we are also able to compare the differences in staining redistribution (ΔFTC) between different samples by taking the difference between respective ΔFTC densities to compute the ΔSP density (Fig 3B). These methods enable visualization of the influence of ABCG2 inhibition on staining while preserving the quantitative aspects of staining intensity and density.

We analyzed the influence of tert-butylhydroquinone (tBHQ) on ABCG2 activity and SP size using an imaging cytometer using these data analysis steps. Following 48 hours of 50 μM tBHQ treatment, A549 cells (4 days post-passage) were found to have and increased SP size from 6% to 9% (S7 Fig). The red signal within the ΔFTC plots reflected the presence of the SP in each sample; similarly, the red signal within the ΔSP plot reflected the increased SP size in the tBHQ-treated sample compared to the control (Fig 3C). Comparing differences in the TGFβ–treated samples shown in Fig 2 (S8 Fig), we observe decreased staining present in the–FTC condition without arbitrary segregation SP and NSP subpopulations from a continuous staining distribution.

### Modeling Transporter Heterogeneity in an *In Silico* Population

We developed an approach for modeling of the SP assay to investigate the role of transporter heterogeneity within the cell population on the generation of SP and NSP responses of individual cells. At the cellular scale, we considered the influence of transporter activity in a mass-action kinetic model to simulate Hoechst transport dynamics and cell staining. At the population scale, we considered the role of heterogeneity in the cell population, defined by variation in transporter properties and cell morphology. We defined an ensemble as the pairing of a particular set of kinetic constraints with all of the staining simulations for an *in silico* population. Having sampled *M* = 10,000 sets of kinetic rate constants (*K*), we have then had 10,000 ensembles with *in silico* staining results that we then compared to our experimental data to assess the ensemble for a SP response at the population level. We then analyzed the influence of transporter properties on staining results at the single-cell level for ensembles demonstrating a SP response.

**Fig 4:**
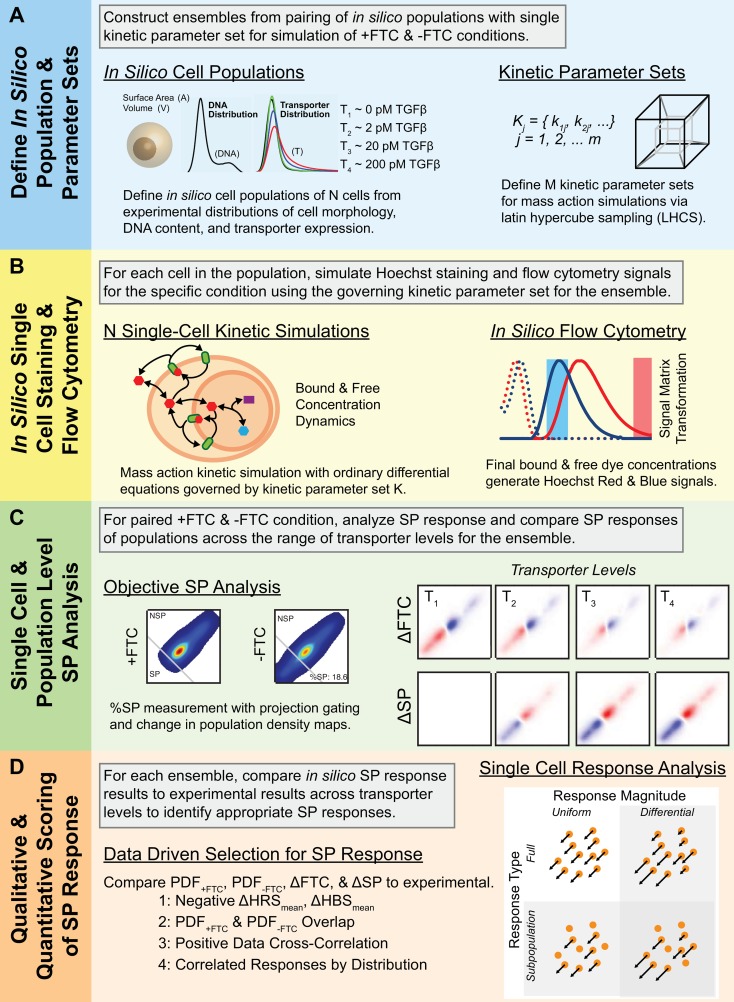
Multiscale Ensemble Approach to Modeling SP Responses. (A) First, a heterogeneous in silico cell population was generated from experimental distributions, which was then paired with each of the kinetic parameter sets that were derived from Latin hypercube sampling of parameter space. The cell population was implemented in 4 different transporter-variant versions, where relative transporter expression was derived from experimental data. An ensemble is defined as a pairing of a particular parameter set with the variety of populations for which it is simulated. Three different models of transporter activity heterogeneity were implemented. (B) For each ensemble, each of the 4 transporter-variant populations were underwent simulated Hoechst staining at the single-cell level both with (+FTC) and without (-FTC) transporter inhibition. Using in silico flow cytometry, the Hoechst concentrations following kinetic staining simulation were converted into Hoechst Red and Blue signals using a linear transformation that incorporates spectral excitation and emission properties of simulated Hoechst dye and flow cytometers. (C) In silico flow cytometry signals were converted into Hoechst Scores and Hoechst Score PDFs. Projection gating objectively assess SP size. Hoechst Score PDFs were used to measure ΔFTC and ΔSP distributions. (D) Hoechst staining metrics of Scores, PDFs, ΔFTC, and ΔSP were used to identify models exhibiting SP responses by comparison to corresponding experimental metrics. Ensembles meeting qualitative selection criteria are then scored according to their similarity in %SP to experimental data. Ensembles demonstrating SP responses were then analyzed on a single-cell level and the distribution of single-cell SP responses analyzed.

Each of the cells in the *N* = 1,000 cell *in silico* population was uniquely defined by parameters for cell volume, cell membrane surface area, nuclear volume, nuclear membrane surface area, DNA content, and transporter heterogeneity (Fig 4A). Morphological parameters for each cell were sampled from corresponding experimental distributions using Latin hypercube sampling (LHCS), which ensured that the resulting *in silico* population faithfully represented the experimental distributions (S9 Fig and S10 Fig). Staining of the *in silico* population was carried out for each individual cell under 4 different levels of transporter expression, T_1_-T_4_, corresponding to distributions derived from 0, 1, 10, and 100 pM TGFβ-treated cells (S10 Fig), under +FTC and–FTC conditions. Single-cell staining simulations consisted of a compartmental mass-action kinetic model of intracellular dye transport processes, governed by kinetic rate constants for the given ensemble and the morphologic parameters associated with the cell (Fig 4A and Fig 1C). Following simulation of each of the cells in a population, the final dye concentrations of the samples were processed via *in silico* flow cytometry to determine Hoechst Red and Blue signals (Fig 4B and S11B, S11C and S11D Fig). Simulated +FTC and–FTC conditions were used to measure SP size, PDF_+FTC_, PDF_-FTC_, ΔFTC, and ΔSP distributions for each of the 4 transporter expression levels (T_1_-T_4_; Fig 4C), which were then compared to corresponding values from the experimental data (Fig 4D). Ensembles were determined to have an SP response if each of the following criteria were met: 1) the change in mean Hoechst Red Signal (ΔHRS_mean_) and Hoechst Blue Signal (ΔHBS_mean_) were negative when comparing the–FTC to the +FTC condition (indicating decreased staining in the–FTC condition); 2) PDF_+FTC_, PDF_-FTC_ distributions overlapped (indicating the presence of a NSP); 3) the cross-correlation between experimental and *in silico* ΔFTC and ΔSP distributions was positive (indicating similarity in spatial response distributions); and 4) *in silico* SP sizes correlated with experimental SP sizes for the 4 transporter expression levels (indicating a consistent decrease in SP size with decreasing transporter expression levels). Ensembles producing a SP response were further analyzed at the single-cell level to investigate the influence of transporter properties on SP/NSP status and differential staining the +FTC and–FTC conditions.

We independently implemented 3 different modes of transporter heterogeneity within the *in silico* cell population to assess the role of transporter heterogeneity in the formation of SP responses in the SP assay. In the first mode of transporter heterogeneity (I), the concentrations of transporter across the population is uniform, which due to the heterogeneity in cell size, leads to a distribution of transporter numbers within the population. In the second mode of heterogeneity (II), the number of transporters per cell across the population is uniform, generating a distribution of transporter densities within the population. For the first two modes, uniform number and densities of transporters for the T_1_-T_4_ expression levels were determined by mean values of ABCG2 expression from flow cytometry data (Fig 2B). We explicitly designed the third mode of heterogeneity (III) to be more expansive than the first two modes. In this mode, transporter expression within the populations were sampled from experimental distributions of ABCG2 expression (S10A Fig) as part of the LHCS approach in generating the *in silico* population. Further, we enabled a non-linear relationship between transporter expression levels and transporter activity levels at the single cell-level. This mimics cooperativity in which higher transporter levels led to greater transporter activity than would be accounted for by a simple 1-to-1 correspondence between expression and activity. We implemented this modeling approach to assess the relationship between transporter heterogeneity and SP responses for the 3 modes of transporter heterogeneity.

### Heterogeneity of Kinetic Transport Processes Generate SP Responses

In our modeling approach, the primary aim was to identify the ensembles in which an SP response resulted. Again, an ensemble consisted of the simulated staining of the *in silico* cell population using a specific set of governing kinetic rate constants (*K*_*j*_) for a particular mode of transporter heterogeneity in both +FTC and–FTC conditions for each of the T_1_-T_4_ transporter expression levels. For each mode of heterogeneity, we assessed *M* = 10,000 kinetic rate constant sets (*K*) for an SP response with less than 5% of the simulation ensembles aborted due to timeout for long simulation time and with SP responses only identified in a small subset, ~5%, of successfully completed ensemble simulations (S15 Fig). In addition, the range of SP responses for each of the modes exhibited a similar quality of fit to experimental SP size (S16 Fig) and a wide range of kinetic rate constants were permissive of SP responses (S17 Fig). An increased frequency of ensembles with SP responses was observed in the concentration (I) and number (II) modes of heterogeneity with larger transporter expression slopes (*k*_*8*_; S17A and S17B Fig), in which a larger *k*_*8*_ value corresponds to greater discrepancy in expression between T_1_-T_4_ conditions. Similarly, in the experimental distribution mode of heterogeneity (III), an increased frequency of SP responses resulted in ensembles with greater non-linearity (*k*_*8*_, *k*_*9*_; S17C Fig). Having met the selection criteria, ensembles exhibiting an SP response were analyzed at the single-cell level for features.

**Fig 5:**
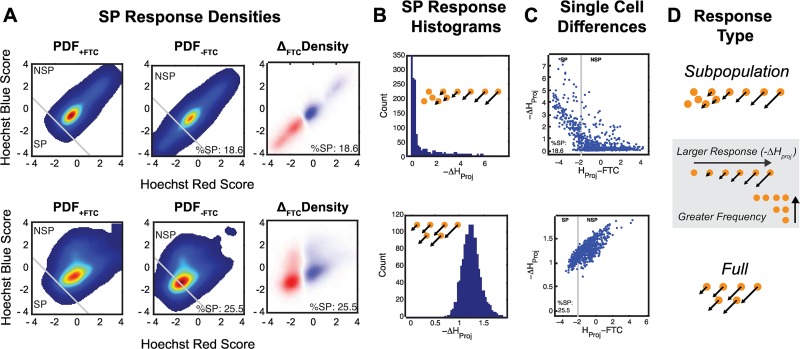
Simulated Staining Population Distributions and Single-Cell Responses. Output from ensembles producing a Subpopulation (above) and Full (below) response are shown. (A) Population densities and differences in staining between simulated +FTC and–FTC conditions demonstrate SP responses and can be compared to corresponding experimental conditions for the untreated control (S1 Fig & S2 Fig). (B) Histograms of the change in staining between +FTC and–FTC conditions for single cells. (C) Plot of the change in staining between +FTC and–FTC conditions for single cells (y-axis) compared to the staining intensity in the–FTC condition (x-axis), shown with the SP and SP gate boundary set by projection gating. (D) Schematic of relative frequencies of single-cell -ΔH_proj_ distributions for Subpopulation and Full response types.

Experimentally, SP responses are apparent when a subpopulation of cells stains less intensely in the–FTC condition compared to the +FTC condition. Using our ensemble modeling approach, we were able to simulate SP responses in heterogeneous *in silico* cell populations. The simulated flow cytometry data generated during the simulations was analyzed using the same approaches for experimental SP data, yielding outputs of SP size, PDF_+FTC_, PDF_-FTC_, ΔFTC, and ΔSP distribution data for the T_1_-T_4_ conditions (Fig 5A). We observed subtle differences in the staining patterns of SP cells, in which some responses exhibit relatively fewer SP cells with a more significant decrease in cell staining (subsequently defined as a *Subpopulation Response*). In contrast, other responses had more SP cells with a less significant decrease in cell staining (subsequently defined as a *Full Response*) between +FTC and–FTC conditions. Experimentally, such differences could only be investigated at the population level due to technical limitations; however, we designed our modeling approach to circumvent these limitations.

A key advantage to our modeling approach was in the consistency of the cell population for staining simulations, in which an identical cell population could be seeded for both +FTC and–FTC conditions. Furthermore, the same *in silico* population was consistent within an ensemble, with the exception of transporter expression by T_1_-T_4_ conditions, and from ensemble to ensemble, differing only by the respective set of governing kinetic rate constant sets. This permitted a level of comparison that would be impossible in an experimental setting. For example, we were able to reduce Hoechst Red and Blue scores onto an x-y projection, as is done in the projection gating method, to obtain a single staining metric for each cell. Because the same in silico cell population was used for both +FTC and–FTC conditions, we were then able to compute the difference in Hoechst staining projection scores (-ΔH_proj_) on a cell-by-cell basis. A larger -ΔH_proj_ corresponds to a greater decrease in staining in the–FTC condition compared to the +FTC condition, thus indicating a greater influence of transporter activity on staining intensity.

Next, we investigated the relationship between the distribution of single-cell -ΔH_proj_ responses with a population and the staining pattern of SP cells for a given ensemble. In plotting histograms of -ΔH_proj_ values we were able to appreciate patterns in responses (Fig 5B). In one pattern, we observed a -ΔH_proj_ distribution with the greatest number of -ΔH_proj_ values near zero and a tail of increasing -ΔH_proj_ values (Fig 5B top). This indicates that transporter inhibition had little to no effect on staining in a majority of the cells but greatly influenced staining in a portion of the cells. We described such a scenario as a *Subpopulation Response*. Contrastingly, in the *Full Response*, the entire population exhibits non-zero -ΔH_proj_ values and a more normal-like distribution (Fig 5B bottom), indicating that staining of each cell in the population was affected by inhibition of transporter activity as well as consistency in the magnitude of this effect.

The nature of our modeling approach enabled us to characterize a great number of relationships not possible experimentally. We compared the influence of transporter inhibition on staining intensity (-ΔH_proj_) against staining intensity in the–FTC condition (H_proj_-FTC; Fig 5C). In the *Subpopulation Response*, we observed SP cells with little influence of transporter inhibition on staining as well as NSP cells with significant influence of transporter inhibition (Fig 5C top). In the *Full Response*, we observed NSP cells with greater influence of transporter inhibition on staining (Fig 5C bottom). Similar comparisons were made to +FTC conditions and by transporter numbers (S19 Fig).

While both were capable of generating SP sizes consistent with experimental data, the *Subpopulation Responses* (S13 Fig) and *Full Responses* (S14 Fig) differed. In the *Subpopulation Response*, the range of staining differences, apparent in the ΔFTC distribution, varied across transporter expression levels (S13D Fig), which was similar to experimental observations (ΔFTC; S8D Fig). However, in the *Full Response*, the range of staining in the (S14 Fig) ΔFTC distributions was consistent across the range of transporter expression levels, varying in intensity (S14 Fig). From a kinetic perspective, decreasing transporter levels in the *Subpopulation Response* was associated with a reduction in the portion of cells influenced by transporter inhibition (S18A Fig) while in the *Full Response* it was associated with a decrease in the magnitude and variability of the -ΔH_proj_ magnitude within the population (S18B Fig).

**Fig 6:**
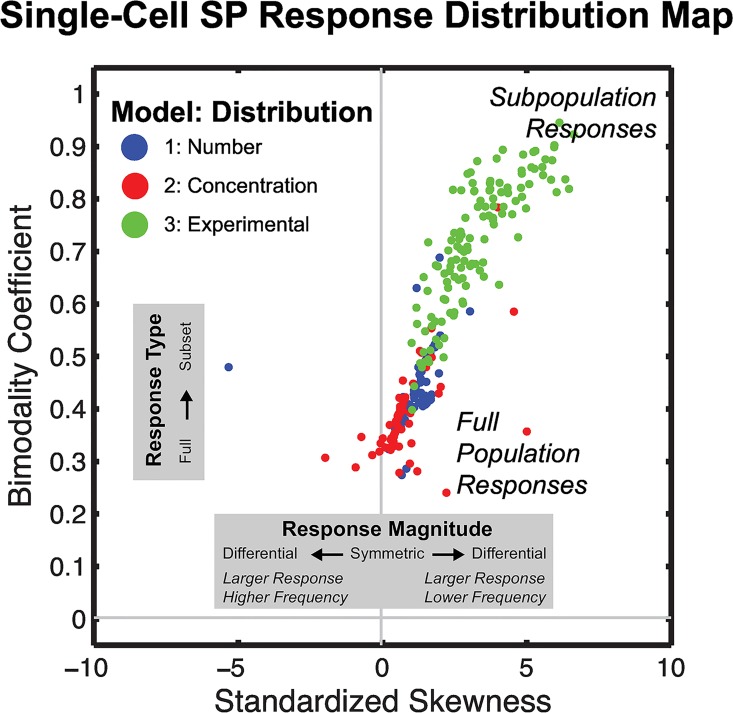
Characterization of Single-Cell SP Response Distributions. The distribution of single-cell SP responses (-ΔH_proj_) of each ensemble exhibiting a SP is depicted by its standard skewness and bimodality coefficient. Distributions from each model are plotted according to their asymmetry along the x-axis (standardized skewness) and similarity towards bimodality along the y-axis. Subpopulation responses had both relatively high bimodality coefficients and standardized skewness while Full responses had low bimodality coefficients and standardized skewness. A qualitative interpretation of the response distribution map is shown in S20 Fig.

For most ensembles, the -ΔH_proj_ response distributions were not clearly *Subpopulation* or *Full Response* in nature, rather most exhibited aspects of both. To more objectively characterize the -ΔH_proj_ responses, the distributions were evaluated by standardized skewness and bimodality coefficient (Fig 6). Standard skewness is a metric that quantifies asymmetry of the distribution while the bimodality coefficient quantifies the similarity between the distribution and a purely bimodal distribution (S20 Fig). Both transporter number (I) and concentration (II) modes of heterogeneity had a greater tendency to generate -ΔH_proj_ distributions more closely resembling the *Full Response* with greater distribution symmetry and bimodality coefficients similar to a normal distribution (blue and red; Fig 6). Ensembles with SP responses generated in the experimental distribution of transporter heterogeneity (III) were more varied, ranging from *Full Responses* to *Subpopulation Responses* (green; Fig 6). The overall spread of distribution mappings in this plot demonstrates a consistency of SP responses arising from -ΔH_proj_ distributions that are roughly normal and have a right-sided tail of variable magnitude.

## Discussion

### Kinetic Determination of SP Response

SP cells arise through ABC transporter activity; however, the technical limitations that prevent staining of specific cells in both inhibitor-free and inhibitor-containing conditions have prevented direct measurement of the influence of transporter activity on Hoechst staining a the single-cell level. Our novel computational approach to *in silico* staining of a cell population in numerous conditions, differing only by transporter activity, revealed that specific distributions of kinetic transport heterogeneity within a population can alone account for the formation of a SP, independent of genetic or other phenotypic traits, such as CSC. This biological insight was obtained by the pairing of flow cytometry distribution data with a novel computational approach for modeling dye kinetics at the single-cell level. This builds on similar approaches by You et al that demonstrated cell-to-cell variability and heterogeneity in gene expression can govern biphasic responses to extracellular cues [27] and that variability of bacterial uptake by hosts are governed in a probabilistic manner determined by host receptor expression levels [28]. It is increasingly understood, even within clonal/isogenic populations, that noisy or variable gene expression leads to heterogeneity within a population and can contribute to differences in phenotype [29–32]. A role for noisy gene expression in generating diverse phenotypic responses in clonal population has been supported both experimentally and computationally; further, it has been postulated as a mechanism of “bet hedging”, thereby conferring survival advantages in stressful or alternate environments [33–37].

The kinetic aspects involved in the SP assay caused us to more carefully consider the significance of SP/NSP discrimination. SP/NSP phenotype are not strictly defined by inheritance or genetic factors, as is indicated by the fact that SP and NSP cells emerge in clonal populations derived from single-cell sorting (S5 Fig) and by reports that describe the interconversion between SP and NSP phenotypes from isolated cell populations [20,38,39]. Further, in our modeling, we observed a range of transporter activities in SP and NSP cells (Fig 5C), suggesting that not all SP cells have high transporter activities and not all NSP cells have low transporter activities. This can be attributed to the interplay between heterogeneity in transporter activity within the population as well as relative differences in DNA content across the population, which alters that staining potential in the SP assay [40]. Thus, the SP phenotype of a specific cell is not discretely defined by membership in a homogenous subpopulation; rather it is a kinetically defined property, inextricably defined by the specific experimental conditions.

### Variability in SP Size

We observed SP sizes ranging from 2% to 20% in the A549 cell line (Fig 2D), which is consistent with reported SP sizes of 2.5% to 30% for A549 cells in the literature [21,38,41–45]. We observed consistent measures of SP size with reproducible trends with increasing time/passage in culture and decreasing exposure to TGFβ (Fig 2D). It should be noted that SP size is determined by the specific experimental conditions in the SP assay, the analytic technique employed by the investigators [1,46–48], and the conditions in which samples are maintained in culture [39,45]. Additional variability may arise from discrepancies in gating SP/NSP regions in cytometry data [21,38,41–45], which is highly subjective and inconsistent and complicated by the fact that SP and NSP staining regions are continuous with no self-evident border of separation (S6A and S6B Fig).

Proper gating to exclude erroneous events is key to accurate SP measurement as cellular debris or dead cells may be mistaken as SP cells. Apoptotic cells, for instance, lie near the SP staining region with greater decreased Hoechst red signal than Hoechst blue signal [1]. Failure to properly exclude these events could result in misclassification of this population as SP when using manual gating methods thereby falsely elevating SP size. The automated SP measurement we described provides an objective measure of the SP without user bias; however, the reliability of the output, %SP, is highly dependent on the input, Hoechst red and Hoechst blue intensities of cytometry events. To ensure the quality of our input we sequentially excluded cellular debris, multiple events, and dead cells in the gating tree to define the final Hoechst Red and Hoechst Blue stained cells as recommended by Golebiewska et al. [1]. Further method development may be necessary to consider scenarios with large apoptotic populations in order to properly exclude such events from the final analysis.

Encouragingly, the automated methods we have described may have potential to measure SP size despite the presence of apoptotic cells as these cells would be expected to stain similarly in -inhibitor and +inhibitor conditions. While an apoptotic population may underestimate SP size by falsely lowering the 1^st^ percentile level in the projection gating method, the staining differences between +inhibitor and -inhibitor conditions would be negated in the ΔFTC distribution. Therefore, the Hoechst staining density distributions may be further developed for SP measurements. While automated methods hold promise for objective measurement of SP size, we emphasize that additional studies will be necessary to establish validity of such measurements in the presence of errant populations.

Variability in biological, experimental, and analytical conditions likely account for the much of the variability in literature-reported SP sizes for the A549 cell line, which highlights the importance of reporting detailed experimental conditions and calls to question the utility of comparing SP size in different experimental and analytical conditions. For example, SP size in untreated A549 cells 4 days after passage had SP sizes of 20% (Fig 2D) and 6% (S7C Fig) when measured using a BD LSR II flow cytometer or Amnis FlowSight imaging cytometer, respectively, despite having identical Hoechst staining conditions and SP data analysis. Instrument settings such as excitation, emission, Hoechst Red and Blue channel windows, detector sensitivity, and detector scaling all greatly influence the recorded Hoechst signals.

### Caveats to the Kinetic Interpretation of SP/NSP Identity

The kinetic interpretation of the SP may be applied to some immortalized cell lines, such as the one used in this study; however, SP may not arise strictly from kinetic properties in all situations, especially in samples of multicellular composition in situ. For example, in the original description of the SP, the SP corresponded to hematopoietic stem cells within bone marrow aspirate [2] and the SP in glioblastoma-derived samples were tumor stromal cells while the glioblastoma cells, including glioblastoma CSCs, did not contribute to the SP [49]. Our investigation adds to this body of evidence as it validates an ABCG2-dependent kinetic basis for the formation of the SP and provides a novel perspective on the distribution of single-cell ABCG2 activity across a population as well as within the SP itself.

Independent of cell line, SP size is also a function of passage frequency, Hoechst staining conditions, fluorescence excitation/emission settings, and SP/NSP gating strategies. Therefore, to focus our investigation on the kinetic aspects of the SP assay, we performed the SP assay with attention to consistency of the culture conditions, Hoechst staining conditions, cytometry settings, and unbiased SP analysis. While this approach enabled investigation of assay kinetics based on differences in ABCG2 expression, other kinetic factors, such as Hoechst staining concentration and duration [46,48], influence SP measurement. A more refined and comprehensive modeling approach would be necessary to determine precise kinetic rate parameters to more definitively characterize the transport phenomena generating the SP.

### The SP Phenotype: Heterogeneity in Therapeutic Targeting

The SP has been the subject of many investigations, many with conflicting results. Some studies claim the SP to be a population of CSCs [20,50], or that ABCG2 is necessary to maintain a stem cell phenotype [51]. In contrast, other studies have identified stem cells lacking a SP phenotype or ABCG2 expression [49,52–54] or identified SP lacking stem cell phenotypes [55–58]. In A549 cells, TGFβ simultaneously leads to enhanced expression of CSC properties [38,43,59] and decreased SP size (Fig 2B) [38]. These findings suggest that SP and CSC phenotypes are nonequivalent; however, the phenotypes appear to be frequently co-expressed.

The co-expression of SP and CSC phenotypes may have a significant role in tumorigenesis as ABCG2 expression can confer a MDR phenotype to CSCs [60]. Additional cellular processes, related to ABCG2 transporter activity, may contribute to resistance in the MDR phenotype. For instance, activity of Nrf2, a master regulator of the cellular redox environment is also a key regulator of ABCG2 expression [61–64]. ABCG2 has been implicated in antioxidant processes [65–71] and TGFβ signaling, down-regulates ABCG2 expression (Fig 2B) in addition to down-regulating multiple antioxidants [38,72–76]. Furthermore, population-level studies have demonstrated increased antioxidant expression in SP cells compared to NSP cells. [77] Therefore, the intracellular redox processes may act in concert with ABCG2-mediated drug efflux to promote the MDR phenotype. Targeting functional properties specific to high ABCG2-expressing cells may be a promising approach to overcome a MDR phenotype. For instance, addition of the tyrosine kinase inhibitor axitinib to topotecan enhanced topotecan-mediated apoptosis in A549 cells through inhibition of ABCG2-mediated transport, independent of ABCG2 expression. [78] Despite the fact that numerous chemotherapeutics are substrates of ABC transporters, evaluation of ABC transporter inhibitors in clinical trials has failed to demonstrate added benefit due to drug toxicities and the inability to achieve sufficient concentrations to effectively inhibit transporters [79]. Further investigation of the kinetic processes involving ABCG2 will refine our understanding of the functional consequences of ABCG2 activity in cancer cells and potentially inspire novel chemotherapeutic approaches.

## Conclusion

This investigation is the first to demonstrate the kinetic mechanisms that form the basis of the SP assay. We validated the ABCG2 transporter-mediated differential staining between +FTC and–FTC conditions across multiple transporter concentrations in a heterogeneous cell population, accounting for differences in responses for SP and NSP cells. Our modeling approach leveraged these experimentally determined dynamic distributions of ABCG2 expression levels as a function of TGF-β treatment and culture time. The computational model tested influences of transporter properties on cell staining across a heterogeneous population, which would otherwise be impossible to achieve due to the technical limitations of the SP assay. Our results suggest that in particular distributions of transporter kinetics within a population, a subset of cells within the population exhibit marked enhancement of transporter activity compared to the main cell population. Analysis of thousands of single cell simulations provided unique insight that NSP and SP cells both lie along a spectrum of ABCG2 activity. Collectively, these results support our hypothesis that specific single-cell distributions of ABC transporter activity yield differential staining and serve as a kinetic basis in forming side populations.

## Materials & Methods

### Cell Culture & Treatment

A549 lung carcinoma cells were obtained from American Type Culture Collection (ATCC; CCL-185) and maintained in growth media, consisting of high glucose DMEM with L-glutamine (Sigma D5796), 10% FBS (Sigma F4135) and penicillin (50 IU/ml)-streptomycin (50 μg/ml) (Cellgro 30-001-CI). Cells were plated in flasks at density of 3,000 cells per well in growth media (15 ml per T-75/35 ml per T-175) and maintained at 37°C and supplemented with 5% CO2. TGFβ (Millipore, GF111) and tBHQ (ACROS Organics, tert-butylhydroquinone, AC15082) treatment took place in culture media.

### Side Population Assay

Hoechst staining and flow cytometry to measure the SP in A549 cells closely followed the protocol described in [23,48]. Cells were trypsinized and resuspended in CO2 conditioned DMEM+, consisting of high-glucose DMEM without phenol red, 10 mM HEPES, 2% FBS, and 2 mM EDTA, at a concentration of 1x106 cells/ml. Samples were split into +FTC and -FTC conditions, supplemented with DMSO-mobilized FTC (EMD Millipore) at a final concentration of 10 μM or DMSO alone, respectively. The solutions were incubated in a 37°C water bath for 30 minutes, after which they were supplemented with Hoechst 33342 (Life Technologies) at a final concentration of 5 μM for 90 minutes, with mixing at 30-minute intervals. The staining solutions were then centrifuged at 1,000 RCF for 10 minutes and resuspended in HBSS+, consisting of HBSS without phenol red, 10 mM HEPES, 2% FBS, and 10 mM EDTA. Cells were incubated with the viability stain SYTOX Blue (Life Technologies, 1:1000) for 5 minutes prior to fluorescence measurement via flow cytometry. Positive controls for dead cell staining were obtained by incubating cells at 56°C for 45 minutes followed by SYTOX Blue staining. The BD LSR II flow cytometer was monitored using fluorescent beads to ensure optimization of system optics and detectors for quality control in polychromatic settings. Samples were excited with a 355 nm UV laser and the Hoechst Red signal was measured in a λem = 675/50 nm channel with linear scaling and the Hoechst Blue signal measured in a λem = 450/50 nm channel with linear scaling, collecting 100,000 events per flow sample. The Hoechst stained -FTC condition was initially used to tune Hoechst Red and Hoechst Blue photomultiplier tube settings, which were held constant for all subsequent studies. Additionally, samples were excited with a 445 nm violet laser with SYTOX blue emission measured in a λem = 473/10 nm channel with logarithmic scaling. Sample gating proceeded as follows: 1) Debris exclusion with FSC-area/SSC-area (λex = 488 nm) gating; 2) Single-cell selection with FSC-height/FSC-area; 3) Live cell selection with the violet-λem = 473/10 nm channel. Events retained through all 3 gates were used for subsequent SP analysis in the Hoechst Red and Blue channels. Manual selection of SP gates was determined using the +FTC conditions where a quadrant gate was placed as tight as possible such that greater than 99% of the cells in the +FTC condition were located in the upper right quadrant. The same gates were then applied to the -FTC condition where the two left gates were considered to be SP gates and the right two gates considered to be NSP gates. The measured %SP in the manual gating approach is the sum of the percent of parent population in the SP gates. For a given sample, the %SP was determined using the specific +FTC and -FTC conditions for that sample.

### Surface Marker Analysis

Following 4 days of TGFβ treatment, surface marker expression of A549 cells was analyzed by flow cytometry following dissociation from culture flasks using non-enzymatic means. Following treatment, cells were dissociated with Enzyme Free Dissociation Solution (Millipore S-004-B) and resuspended in DMEM without phenol red supplemented with 2% fetal bovine serum, 10 mM EDTA, and 10 mM EGTA. The cells were then pelleted (1000 RCF, 10 minutes) and resuspended at a concentration of 1x107 cells per ml in HBSS without phenol red, Ca2+, & Mg2+ supplemented with 1 mM HEPES, 2% fetal bovine serum, 1 mM EDTA, and 1 mM EGTA. The cell solution was added to an equal volume of antibody staining solution and incubated for 30 minutes on ice with gentle rotation. Antibody solutions (mouse IgG_1_ anti-E-cadherin/PE-CF594, BD Biosciences, Clone 67A4, 2x dilution; mouse IgG_1_ anti-N-cadherin/PE, BD Biosciences, Clone 8C11, 2x dilution; mouse IgG_2_ anti-ABCG2/APC, BioLegend, Clone 5D3, 2x dilution) were prepared in the aforementioned HBSS solution. Following incubation, cells were washed and resuspended in the HBSS solution. Next the solutions were stained with SYTOX Blue to select for live cells. A BD LSR II flow cytometer was used to analyze fluorescence of the stained cells with the following settings: FSC/SSC (λex = 488 nm), PE (λex = 488 nm, λem = 575/26 nm), PE-C594 (λex = 488 nm, λem = 610/20 nm), APC (λex = 633 nm, λem = 660/20 nm), SYTOX Blue (λex = 445 nm, λem = 473/10 nm). Unstained and single-stained control samples were prepared to determine compensation matrix corrections within FlowJo for each replicate. Heat killed control cells were mixed with live cells to establish live-dead discrimination with SYTOX Blue staining. We employed the following gating strategy: debris exclusion (FSC/SSC), single events (FSC-H/FSC-A), and live cells (SYTOX Blue). Upon gating for single, live-cell events, fluorescence intensities were measured as the geometric mean fluorescence.

### Hoechst Staining Signal Processing

#### Hoechst Score Transformations

Side population flow cytometry data for a particular sample consists of individual events with associated Hoechst Red and Blue signals for both +FTC and -FTC conditions. The Hoechst Red and Blue signals are expressed in independent arbitrary units. To generalize the interpretation of side populations from Hoechst signals, independent of raw signal units, Hoechst signals were converted into Hoechst Scores. Hoechst Scores are based upon the standard score, or z-score, in which a distribution is mean-centered and normalized to the standard deviation. To compute the Hoechst Score for the Hoechst Red channel, the mean and standard deviation of the Hoechst Red signal from the +FTC condition were calculated. Next, the +FTC Hoechst Red mean was subtracted from the Hoechst Red signals from each of the events in the +FTC and -FTC conditions. Similarly, each event in the +FTC and -FTC conditions were divided by the standard deviation from the +FTC condition. The resulting event data, +FTC mean centered and +FTC standard deviation normalized, constituted the Hoechst Red Scores for the two conditions. Hoechst Blue Scores were derived in an analogous fashion. Hoechst Scores were calculated at a per sample basis between each pairing of +FTC and -FTC condition data.

#### Projection Gating for %SP Measurement

Hoechst Scores Projections were derived from Hoechst Red and Blue Scores. Projections were derived from Score data and not signal data due to the arbitrariness of signal magnitude in the signal data. Score data from Hoechst Red (HRS) and Blue (HBS) channels have common units and relative magnitudes. Projection values were derived for each event within a sample, in which the Hoechst Score Projection (H_proj_) was defined:

$$H_{proj}=\frac{HRS+HBS}{\left|HRS+HBS\right|}\sqrt{\frac{1}{2}{\left(HRS+HBS\right)}^{2}}$$

Hoechst Projections were used to set a threshold, or gate, intensity at the lower limit of the NSP. The 1^st^ percentile mark of the Hoechst Projection data from the +FTC condition was used define the threshold and applied to the -FTC condition. The percent of events falling below the threshold in the -FTC condition was set as the %SP in this projection gating approach.

#### Hoechst Scores PDF Distributions

For a given Hoechst condition (+FTC or -FTC), Hoechst Red and Blue scores were provided transformed into a 2D probability density function (PDF) on the Hoechst Red and Hoechst Blue plane with the frequency, or cell density, defined at each paired Hoechst Red and Blue coordinate. Smoothed surfaces over the Hoechst Red and Blue plane were derived by the method for smoothing scatter plot data described by Eilers and Goeman [80]. Next, the area under the surface was calculated and normalized to one for each condition.

In its most basic form, flow cytometry data is a set of coordinate data/scatter, with each event represented by an intensity value along each of the measured dimensions. Comparison of data between two sets relies on the comparison of some sort of statistical transformation of the data (i.e. mean or median). However, such transformations result in loss of spatial information. Histograms allow for comparison of spatial information, but differences in event number complicate interpretations. In order to permit more rigorous comparisons, we converted Hoechst staining coordinate data into probability density functions along both Hoechst Red and Blue Score dimensions. This was similar, in effect, to constructing a 2D histogram with smoothing and normalization to account for differences in event number between data sets. In this format, spatial differences in staining distributions were easily computed by taking the difference between PDFs.

#### ΔFTC Distributions

For a given sample, a ΔFTC distribution was calculated by taking the point-wise difference between the PDF of the -FTC condition (PDF_-FTC_) and PDF_+FTC_ across the Hoechst Red and Blue plane to calculate the difference in normalized cell density.

#### ΔSP Distributions

To compare the difference between ΔFTC distributions between two samples we calculated a ΔSP distribution. To compare a test sample to a control sample the ΔSP was derived as ΔFTC_test_—ΔFTC_control_, where the point-wise difference in ΔFTC intensities was calculated at each Hoechst Red and Blue pair.

### FlowSight Imaging Cytometer

We imaged samples at 20X magnification using a FlowSight imaging cytometer with the Quantitative Imaging Upgrade (Amnis, Seattle, WA). Single color controls were used to set-up compensation matrices. For the SP assay, the compensation matrix was manually edited to allow collection of the Hoescht Blue (470/35 nm) and Red (694/51 nm) signals using the 405 nm laser. Images were analyzed with IDEAS analysis software (Amnis). Using the gradient root mean square feature for the brightfield channel, “Focused cells” were selected according to the manufacturer’s recommendation. Debris was eliminated by gating single cells using the area and aspect ratio features for the brightfield channel. Live cells were gated using the intensity feature in the green channel (533/27 nm) for SYTOX Green staining. For ABCG2 analysis, the intensity feature of the APC channel (694/51 nm; 642 nm excitation laser) was used to quantify the expression of ABCG2. For the side population assay, double positive cells from were selected by gating in the Hoechst Red and Hoechst Blue channels.

### Ensemble Modeling of Side Population Responses

#### General Overview of Approach

Heterogeneous cell populations were simulated in an array of experimental conditions across a wide range of kinetic conditions to investigate the influence of transporter function on Hoechst staining kinetics that give rise to SP phenotypes. Three models were generated describing numerical (Model 1), concentration (Model 2), and experimentally derived (Model 3) transporter distributions. Individual cells (P_i_) within a population (P) of size N = 1,000 are described by a set of morphological parameters (volumes, surface areas, & DNA content, Table 1). The parameter values for the population were assigned via LHCS of PDFs derived from experimental distributions of cell radii, nuclear radii, and DNA content. Kinetic parameter sets (M = 10,000) were obtained from LHCS of uniform distributions in log space (Fig 4A). For each parameter set, Hoechst staining is simulated in the cell population across multiple transporter conditions and with and without transporter inhibition, simulating +FTC and -FTC conditions. Each population simulation consists of N single-cell mass-action ODE simulations of Hoechst staining (Fig 4B). Following the kinetic simulations, Hoechst concentrations within individual cells were converted to Hoechst Red and Blue signals via linear transformation with a signal matrix accounting for the spectral excitation and emission properties of free and DNA-bound Hoechst dyes as well as an in silico flow cytometer (Fig 4B). Simulated flow cytometry signals, in arbitrary units, were then converted to Hoechst Score PDFs where projection gating was applied to measure the %SP (Fig 4C). Hoechst scores PDFs were used to calculate ΔFTC and ΔSP distributions, allowing for visualization of differences in population simulations with and without transporter inhibition as well as across different transporter conditions (Fig 4C). Hoechst score metrics from in silico flow cytometry populations were compared to metrics derived from experimental data to gauge the extent of SP response in the populations. Parameter sets with in silico populations meeting the selection criteria for identifying a SP were then accepted and ranked according to similarity to %SP measured experimentally according to the normalized root mean-squared error (RMSE, Fig 4D). Accepted sets were then analyzed at the single-cell SP response. The distribution of responses to inhibition were used to classify the homo/heterogeneity of response magnitudes and the uniformity/bimodality of response frequency (Fig 4D).

**Table 1:** Single-Cell ODE Model Morphology & Expression Variables. The variables listed below were assigned by LHCS of experimental distributions for each cell within an *in silico* cell population.

Parameter	Symbol	Units
Cytosolic Volume	*V*_*C*_	*pl*
Plasma Membrane Surface Area	*A*_*C*_	μ*m*^2^
Nuclear Volume	*V*_*N*_	*pl*
Nuclear Surface Area	*A*_*N*_	μ*m*^2^
Relative DNA Level	*DNA*_*L*_	*DNA*_*Level*_
Relative Transporter Level	*T*_*Total*_	*T*_*Level*_

#### Whole Cell and Nuclear Radii Probability Density Functions

FlowSight imaging cytometry was used to measure whole cell and nuclear radii of Hoechst stained cells in the presence of FTC as per the imaging cytometer instructions. Cells and nuclei had aspect ratios > 0.9 and were assumed to be spherical for the purposes of simplification. Whole cell and nuclear areas, reported in micrometers, were then used to estimate cell and nuclear radii for the population of A549 cells (S9A Fig). The size of the nucleus was partially correlated to the whole cell size (S9B Fig); therefore, instead of treating each as in independent distribution, a 2D PDF was constructed in a manner analogous to the used to calculate PDFs for Hoechst Red and Blue Scores.

#### Different Transporter Expression & Distributions

Transporter Expression: Different models of transporter distribution across a population were implemented; however, at the single-cell level, the Hoechst staining kinetic model was identical. Therefore, what differed between the models was how different cells within a given population were assigned transporter expression. In each of the models, experimentally-derived expression levels were used to inform model expression. In Models 1 & 2, the relative geometric mean expression of ABCG2 from TGFβ -treated A549 cells (Fig 2B) were used while in Model 3, the flow cytometry staining distribution served as a probability density function from which transporter expression frequency was sampled (S10A Fig).

Model 1. Number Distribution/Equal Concentration:

Each population was associated with the geometric mean as the relative transporter level for the population. This factor served as the scaling factor relative to the maximum geometric mean intensity from the untreated control condition. The magnitude of the relative differences between transporter levels of the samples was set by *k*_8_. The relative differences of transporter levels were reconfigured for each kinetic parameter set. Once the relative transporter level was determined, it was set as the maximum transporter concentration in the cytosol or nucleus where the relative intensities of cytosolic to nuclear transporter activities were expressed as:

$$T_{CTA}=k_{7}T_{NTA}$$

Each cell within the population was assigned total cytosolic and nuclear transporter activities of *T*_*CTA*_ & *T*_*NTA*_, respectiviely.

Model 2. Concentration Distribution/Equal Number:

Transporter activity for Model 2 proceeded in the same manner as Model 1; however, after calculation of *T*_*CTA*_ & *T*_*NTA*_, the average number of molar equivalents in cells of the distribution was calculated. Each of the cells in the population were then assigned the same number of molar activity equivalents, which was then converted to concentration activity equivalents on a cell-by-cell basis using the cytosolic and nuclear volumes.

Model 3. Experimental Distribution:

Flow cytometry ABCG2 surface marker staining data from TGFβ -treated A549 cells were exported from FlowJo as compensated fluorescence intensities. The distributions were then loaded into MATLAB where they were converted to PDFs for each individual replicated. PDFs were generated with the *ksdensity* function. For a particular sample, a final PDF was taken as the unit normalized average PDF of three experimental replicates. The distributions were then scaled to fall between 0 and 1 (S10A Fig). Therefore, values within the distribution reflect relative expression within the distribution.

Relative transporter levels within a distribution (*T*_*Total*_) were randomly selected values (*P*_*Ti*_) from ABCG2 expression PDF distributions where *i* = [1,4], corresponding to ABCG2 distributions from 0, 1, 10, & 100 pM TGFβ treatments, respectively. *T*_*Total*_ levels are expressed in units of *T*_*Level*_. Next, the relative transporter levels in cytosolic (*T*_*CT*_) and nuclear (*T*_*NT*_) compartments were calculated under the assumption that the total transporter level is split into cytosolic and nuclear compartments at a fixed ratio, which remains constant during the simulation.

Total Transporter Level:

$$T_{Total}=T_{CT}+T_{NT}$$

Cytosolic/Nuclear Transporter Levels:

$$T_{CT}=k_{7}T_{NT}$$

Total transporter activity levels were then calculated from the Hill equation (*θ*_*c*_), reflecting the cooperative interactions of transporters within each compartment:

Total Cytosolic Transporter Cooperatively:

$$T_{CTA}=\theta_{c}(T_{CT})$$

Total Nuclear Transporter Cooperatively:

$$T_{CNA}=\theta_{c}(T_{NT})$$

Transporter Cooperatively:

$$\theta_{c}(T)=\frac{T^{k_{8}}}{{k_{9}}^{k_{8}}+T^{k_{8}}}$$

#### Transporter Activity Level Inhibition

Prior to kinetic modeling of Hoechst staining, each cell was assigned a total cytosolic and nuclear transporter activities, *T*_*CTA*_ & *T*_*NTA*_. The absolute transporter activity in the kinetic simulation was then determined by the absolute scaling factor *k*_6_ as well as the degree of transporter inhibition (*i*_*T*_), where *i*_*T*_ = 0.99 in the inhibited condition (+FTC) while *i*_*T*_ = 0 in the uninhibited condition (-FTC).

Total Cytosolic Transporter Activity:

$$T_{CA}=k_{6}{(1-i}_{T})T_{CTA}$$

Total Nuclear Transporter Activity:

$$T_{NA}=k_{6}({1-i}_{T})T_{CNA}$$

#### Hoechst/DNA-Binding Site Expression Probability Density Function

DNA content distributions within cell populations can be measured with Hoechst staining. [81] Therefore we took the +FTC Hoechst stained samples to represent the relative distribution of DNA content within a population. Hoechst Blue signals for each of the +FTC conditions in the SP time course study were loaded into MATLAB and converted into a PDF using the ksdensity function. An overall distribution was constructed from the average of the 39 individual PDFs (S10B Fig). The distribution was then normalized to the mode so that the distribution represented a distribution relative to the mode. The mode was assumed to represent a cell in the G_0_/G_1_ phase and have a relative DNA content of the size of 1 genome for an aneuploid A549 cell. We used this distribution to estimate the number of Hoechst-binding sites in DNA (S10B Fig).

**Table:** 

A549 Genome Size = 7.3x10^8^ Base Pairs	[82]
Base Pairs Per Hoechst Binding Site = 80	[83]
Binding Site Number per Mole (*AvgN*) = 6.022x10^23^	

For a given cell, the relative DNA level sampled as *DNA*_*L*_ with units of *DNA*_*Level*_. Within the population, each of the DNA intensities was identically scaled, though maintaining their relative distribution, to determine absolute binding sites and converted to molar binding sites. Finally, binding site number was factored by nuclear volume to derive a molar concentration of binding site number.

$$DNA_{Total}={DNA}_{L}∙(Genome\;Size)∙(Base\;pairs\;per\;site)∙k_{2}/AvgN$$

#### Sampling PDFs to Construct In silico Cell Populations

To sample PDFs and produce in silico populations, PDFs were converted, approximately, to cumulative distribution functions (CDF) by taking the cumulative sum of a PDF. The CDFs were then normalized to a range of 0 to 1. To generate a population of N cells from the PDF, N random numbers were drawn from the interval of 0 to 1 and mapped to the CDF to find the corresponding expression value. In our observation, random sampling over the entire interval produced highly variable effects. To circumvent this issue, we implemented Latin hypercube sampling (LHCS) of the CDF, which more uniformly sampled the distribution. To sample the radii, Nx2 random numbers between 0 and 1 were generated. The whole cell PDF was sampled as an independent PDF. Next, the nuclear PDF for the given whole cell radii was sampled to sample the conditional PDF for nuclear radii size. Reconstructed cell populations of various sizes are shown in S9C Fig. After the radii were sampled for a given cell, the radii were used to calculate cell and nuclear volumes and surface areas, assuming spherical morphology. Cytosolic volumes were taken as the difference between whole cell and nuclear volumes. Notably, the same LHCS vector was used to sample the different CDFs of transporter expression. Therefore, the sampling of transporters between populations is consistent.

#### Latin Hypercube Sampling (LHCS) of Kinetic Parameter Space

M combinations of kinetic parameters (*k*_*q*_; Table 2) were assigned via LHCS, which segments a dimension of parameter space into uniform segments. Within each segment, a parameter value is selected from a uniform random distribution. Thus, LHCS generates a collection of randomly chosen parameter choices with nearly uniform sampling of the parameter space. Within MATLAB, the LHCSdesign function was used to generate an MxQ LHCS matrix of M samples within the interval (0,1) for each of the L parameters (Models 1 & 2, Q = 8; Model 3, Q = 9). The criterion correlation and maxmin were enabled and 50 iterations were permitted to reduce correlation and maximize point-to-point distance within the LHCS matrix.

**Table 2:** Single-Cell ODE Model Kinetic Parameters. Sets of random kinetic parameters were assigned using LHCS to uniformly sample the parameter range in Log_10_-spaced intervals. The set of kinetic parameters is a uniformly applied across a population.

Parameter	Symbol	Range	Units
Hoechst-DNA Association Rate	*k*_1_	10^−1^–10^5^	1μM∙min
†Hoechst-DNA Dissociation Rate	*k*_*off*_	*k*_1_ ∙ 10^−7^	1min
DNA Binding Site Scaling Term	*k*_2_	10^−3^–10^3^	N/A
Hoechst Membrane Permeability	*k*_3_	10^−6^–10^4^	amolμM∙μm2∙min
Hoechst-Transporter Association	*k*_4_	10^−6^–10^12^	1μM∙min
Hoechst-Transporter Dissociation	*k*_5_	10^−6^–10^12^	1min
Absolute Transporter Expression	*k*_6_	10^−6^–10^6^	μMTLevel
Cyt./Nuc. Transporter Ratio	*k*_7_	10^−5^–10^5^	N/A
* Transporter Expression Slope	*k*_8_	10^−2^–10^2^	N/A
** Transporter Hill Coefficient	*k*_8_	10^0^−10^1^	N/A
** Transporter Half-Maximal Level	*k*_9_	0–1	*T*_*Level*_

* Model 1 & 2

** Model 3

† *k*_*off*_ set according to the value reported by [83]

To convert the LHCS of the parameter space ranges in Log_10_ space for a given parameter *k*_*q*_, the q^th^ column of the LHCS matrix was scaled by the Log_10_ of the range size and increased by Log_10_ of the lower limit of the range. Finally, the parameter value *k*_*mq*_ was obtained by taking the antilog of the m,q^th^ entry of the transformed LHCS matrix. In Model 3, the Hill Half-Maximal Level, *k*_9_, was sampled uniformly from 0 to 1.

Initial modeling included *k*_*off*_ within the parameter search space; however, early interrogation of the system demonstrated insensitivity to variation in *k*_*off*_. Therefore we maintain a fixed *k*_*off*_ relative to *k*_1_ in all ensembles based on the reported *K*_*D*_ of 10^−7^. [83]

Kinetic Model of Hoechst Staining:

Simulation of Hoechst staining took place at the single-cell level. Within each population, cells were assigned variable cell and nuclear sizes, DNA content, and, in Model 3, relative transporter levels. Across the set of kinetic parameters were common across the entire population. Hoechst staining within a single cell was modeled using mass-action kinetics to describe the rates of reaction the transport across plasma and nuclear membranes (S11A Fig). Each single cell system was modeled with three spatial compartments and was simulated with 90 minutes of staining.

In each simulation, cells were initialized with no Hoechst species within the cell. The extracellular compartment was assumed to be so large so as to not experience changes in Hoechst concentration throughout the simulation. We assumed total DNA binding sites, cytosolic transporter, and nuclear transporter levels were conserved during the time course of the staining and, using conservation of mass, we algebraically reduced the order of the system, setting the order of the system at 5 differential variables (Table 3). The set of kinetic reactions were used to compose the set of differential equations, which governed the dynamics of Hoechst-associated species within the kinetic model.

**Table 3:** Single-Cell ODE Model Mass-Action Variables. The variables listed below are the species involved in mass-action kinetic reactions to simulate the staining of cells with extracellular Hoechst.

Species	Symbol	Initial Condition	Variable Type
Extracellular Hoechst	*H*_*e*_	5 μM	Constant
Cytosolic Hoechst	*x*_1_	0	Differential
DNA-Bound Hoechst	*x*_2_	0	Differential
Unbound DNA Binding Sites	*DNA*	*DNA*_*T*_	Algebraic
Total DNA Binding Sites	*DNA*_*T*_	*DNA*_*Total*_	Constant
Hoechst-Bound Cytosolic Transporter	*x*_3_	0	Differential
Unbound Cytosolic Transporter	*T*_*C*_	*T*_*CA*_	Algebraic
Total Cytosolic Transporter	*T*_*CA*_	*k*_6_*T*_*CTA*_	Constant
Nuclear Hoechst	*x*_4_	0	Differential
Hoechst-Bound Nuclear Transporter	*x*_5_	0	Differential
Unbound Nuclear Transporter	*T*_*N*_	*T*_*NA*_	Algebraic
Total Nuclear Transporter	*T*_*NA*_	*k*_6_*T*_*NA*_	Constant

For each single-cell simulation, variables were assigned initial conditions and submitted with system reaction equations to the *ode15s* solver in MATLAB. Constant variables remain unchanged during the course of the simulation. Algebraic variables were derived from constant and differential variables using algebraic conservation equations at each time point in the solver. Differential variables were solved at each time point in the solver according to the set of differential equations.

Mass Conservation Equations:

During a simulation of Hoechst staining in a single cell, the amount of transporter and Hoechst/DNA-binding sites are assumed conserved and un-changed in total quantity. Algebraic terms accounting for this conservation are substituted into the model for simplification and to reduce the order of the model.

Hoechst/DNA-Binding Sites:

$$DNA_{T}=DNA+x_{2}$$

Cytosolic Transporter:

$$T_{CA}=T_{C}+x_{3}$$

Nuclear Transporter:

$$T_{NA}=T_{N}+x_{5}$$

Kinetic Reaction Equations

Plasma Membrane Diffusion:

$$r_{1}=k_{3}\frac{A_{C}}{V_{C}}(H_{e}-x_{1})$$

Hoechst-DNA Association:

$$r_{2}=k_{1}x_{4}DNA$$

Hoechst-DNA Dissociation:

$$r_{3}=k_{off}x_{2}$$

Cytosolic Hoechst-Transporter Association:

$$r_{4}=k_{4}x_{1}T_{C}$$

Cytosolic Hoechst-Transporter Dissociation & Efflux:

$$r_{5}=k_{5}x_{3}$$

Nuclear Membrane Diffusion:

$$r_{6}=k_{3}A_{N}(x_{4}-x_{1})$$

Nuclear Hoechst-Transporter Association:

$$r_{7}=k_{4}x_{4}T_{N}$$

Nuclear Hoechst-Transporter Dissociation & Efflux:

$$r_{8}=k_{5}x_{5}$$

Differential Equations:

Cytosolic Hoechst:

$$\frac{{dx}_{1}}{dt}=r_{1}-r_{4}+\frac{r_{6}}{V_{C}}+\frac{V_{N}}{V_{C}}r_{8}$$

DNA-Bound Hoechst:

$$\frac{{dx}_{2}}{dt}=r_{2}-r_{3}$$

Cytosolic Transporter-Bound Hoechst:

$$\frac{{dx}_{3}}{dt}=r_{4}-r_{5}$$

Nuclear Hoechst:

$$\frac{{dx}_{4}}{dt}=-r_{2}-r_{3}-\frac{r_{6}}{V_{N}}-r_{7}$$

Nuclear Transporter-Bound Hoechst:

$$\frac{{dx}_{5}}{dt}=r_{7}-r_{8}$$

#### In silico Flow Cytometry Simulation

Conversion of simulated Hoechst staining into Hoechst Red and Blue signals was mediated by a linear transformation of DNA-bound Hoechst and non-DNA-bound (free) Hoechst species within each cell in a process we refer to as *in silico* flow cytometry. Following the kinetic simulation of Hoechst staining, the molar quantity of total free Hoechst and DNA-bound Hoechst are determined for each cell. The quantities of these dyes were used to calculate a corresponding Hoechst Red and Hoechst Blue signal. Hoechst Red and Blue signals result from the combination of Hoechst Red and Blue emission from both DNA-bound and free Hoechst species (S11B Fig). DNA-bound and free Hoechst dyes possess different spectral properties, including quantum yield, excitation maxima, and emission maxima. [84] These differences manifest as differences in relative excitation efficiency and emission strength in the Hoechst Red and Blue emission channels (S11C Fig). Accounting for these factors, we are able to formulate a signal transformation matrix with which we can transform quantities of DNA-bound and free Hoechst into relative Hoechst Red and Blue signals.

Most flow cytometry techniques aim to isolate the signal from an individual fluorophore to a single detection channel. The SP assay, however, relies on spectral spillover of the Hoechst emission into two detectors, Hoechst Red and Hoechst Blue. Inherent spectral differences in DNA-bound (*H*_*b*_) and non-DNA-bound/free (*H*_*f*_) Hoechst dyes would indicate that the two forms of Hoechst can influence the detected signal in each of these channels (S11C Fig). For example, the quantum yield of DNA-bound Hoechst (0.38) is roughly 10-fold larger than that of free Hoechst (0.034) [84], and more readily induced to emit fluorescent light upon excitation. Further, the excitation/emission maxima of Hoechst in the DNA-bound form differs from the free form. [84] The differences in emission spectra result in differential emission contributions to each of the detection channels (S11D Fig). In this schema, a number of factors influence the magnitude of the Hoechst Red and Blue emission signals. Nonetheless, it is a somewhat constrained system in that the Hoechst Red signal is composed of emission from both DNA-bound and free Hoechst and the Hoechst Blue signal is composed of emission from both.

Hoechst signals are calculated based upon the quantities of DNA-bound and free Hoechst species within the cell, the spectral properties of the Hoechst species, and the spectral properties of the simulated flow cytometer used to excite and measure Hoechst fluorescence. Hoechst Red signal (*HR*_*sig*_) is the sum of the emission from DNA-bound (*H*_*b*_) Hoechst in the Hoechst Red channel and from free Hoechst (*H*_*f*_) in the Red Channel. The emission from *H*_*b*_ in the Hoechst Red channel is proportional to its excitability (quantum yield, *Q*_*b*_), relative excitation efficiency (*E*_*b*_), the area of spectral emission overlap with the Hoechst Red channel (*R*_*b*_), and the amount of *H*_*b*_. Likewise, the emission from *H*_*f*_ in the Hoechst Red channel is proportional to its corresponding *Q*_*f*_, *E*_*f*_, *R*_*f*_, and *H*_*f*_ (Table 4). Signal for the Hoechst Blue channel can be similarly constructed.

$${HR}_{sig}=Q_{b}E_{b}R_{b}H_{b}+Q_{f}E_{f}R_{f}H_{f}$$

$${HB}_{sig}=Q_{b}E_{b}B_{b}H_{b}+Q_{f}E_{f}B_{f}H_{f}$$

**Table 4:** Spectral Quantities for *In silico* Flow Cytometry Signal Transformation

	Description	Symbol	Value
DNA-Bound Hoechst	Quantum Yield	*Q*_*b*_	0.34
	Excitation Efficiency	*E*_*b*_	0.9902
	Hoechst Red Emission	*R*_*b*_	29.86
	Hoechst Blue Emission	*B*_*b*_	4650
Free Hoechst	Quantum Yield	*Q*_*f*_	0.038
	Excitation Efficiency	*E*_*b*_	0.6764
	Hoechst Red Emission	*R*_*f*_	464.2
	Hoechst Blue Emission	*B*_*f*_	1914

We are then able to modify the representation of to obtain the linear transformation matrix *S*.

$$\left[\begin{array}{cc} Q_{b}E_{b}R_{b} & Q_{f}E_{f}R_{f}\\ Q_{b}E_{b}B_{b} & Q_{f}E_{f}B_{f} \end{array}\right]\left[\begin{array}{c} H_{b}\\ H_{f} \end{array}\right]=\left[\begin{array}{c} {HR}_{sig}\\ {HB}_{sig} \end{array}\right]\;S\left[\begin{array}{c} H_{b}\\ H_{f} \end{array}\right]=\left[\begin{array}{c} {HR}_{sig}\\ {HB}_{sig} \end{array}\right]$$

where

$$\left[\begin{array}{cc} Q_{b}E_{b}R_{b} & Q_{f}E_{f}R_{f}\\ Q_{b}E_{b}B_{b} & Q_{f}E_{f}B_{f} \end{array}\right]=\left[\begin{array}{cc} s_{11} & s_{12}\\ s_{21} & s_{22} \end{array}\right]=S$$

The Hoechst Red and Blue signals resulting from linear transformation with the signal matrix are arbitrary in that the units do not have a specific meaning. Nonetheless, within a range of Hoechst signals produced under the same circumstances, differences in magnitude reflect differences in the quantities of Hoechst dyes used to generate them. Therefore, the Hoechst signals can be compared for staining within populations. Similarly, with identical conditions, comparisons can be made across populations. Conversion of Hoechst signals into Scores is an approach to make measured changes in Hoechst staining more applicable in a broader, less experimentally specific sense.

#### Hoechst Score & PDF Conversion of Flow Cytometry Signals

Following the calculation of in silico flow cytometry values of Hoechst Red and Blue signals for each cell in a population, Hoechst Scores data was derived from Hoechst signal data. Processing of data from the in silico flow data was identical to that of data processed in real flow cytometry data. For a given in silico sample (inhibition and no inhibition pairing), PDF_+FTC_, PDF_-FTC_, and ΔFTC distributions were calculated. The SP size was measured using the projection gating approach. Finally, across the four conditions, the ΔFTC distributions were compared to the ΔFTC distribution from the highest transporter sample, corresponding to untreated control, to calculate ΔSP distributions.

#### Data-Driven Qualitative Selection of SP Responses

Simulation of each of the ensembles produced the following data: differences in Hoechst Scores staining metrics, Hoechst Score PDF+FTC, Hoechst Score PDF-FTC, ΔFTC, and ΔSP relative to the control, and %SP for each in silico sample. In the analogous experimental conditions, Day 4 of the SP time course with 4 differing TGFβ sample conditions, we possess equivalent experimental data (Fig 2D, S3 Fig and S8C Fig). We used the experimental data to guide selection of models in terms of SP response. A series of selection check points were setup, which each ensemble was required to meet all selection criterion in order to be accepted as exhibiting a SP response.

First, the two highest transporter conditions were required to have negative ΔHRS_mean_ and ΔHBS_mean_ values, reflecting an overall decrease in Hoechst staining. Next, the PDF_+FTC_ and PDF_-FTC_ were not allowed to share any less than 25% overlap, indicating that the entire range of population did not shift without inhibition. The responses across all of the transporter conditions were required to reflect that of the experimental data. Ensembles were required to demonstrate a positive correlation for both the ΔHRS_mean_ with experimentally observed ΔHRS_mean_ values and ΔHBS_mean_ with experimentally observed ΔHBS_mean_ values. Experimental PDF_+FTC_, PDF_-FTC_, ΔFTC, and ΔSP distributions for all conditions were exported from the SP time course study. The normalized, aligned 2D cross correlation was calculated for each simulation/experimental pairing. Within each of the categories, PDF_+FTC_, PDF_-FTC_, ΔFTC, and ΔSP, the average cross correlation of all of the conditions was required to be positive. Finally, ensembles were required possess a %SP of at least 5% for the control sample and a differential %SP of 2.5% between the control and lowest transporter expressing sample. Ensembles meeting the selection criteria were then scored according to the normalized root mean-square error (RMSE) of the %SP and differences in %SP with the experimental data using the MATLAB function *goodnessOfFit* with a normalized root mean square error (*NRMSE*) cost function. Fit values using this approach range from 1, with a perfect fit of simulated data to experimental data, to negative infinity with increasingly poor fit to experimental SP data.

#### Analysis of Single-Cell Side Population Response Distributions

Within an ensemble, each of the cell populations is identical to one another, except for the relative amount of transporter activity. Unlike experimental assays, cells within the in silico assay are indexed and differences between samples perfectly controlled for. Therefore, we can examine how the exact same cell will stain differently under very tightly controlled alternate scenarios. Because of this feature, we can tabulate the difference in Hoechst Score projection in the inhibited and uninhibited Hoechst staining simulations. Thus, for each cell in a sample, we determine the difference between these two conditions as -ΔH_proj_, in which a larger value corresponds to a larger single-cell SP response. For each ensemble passing the qualitative selection process, the distributions of -ΔH_proj_ values for each of the samples was further analyzed to interrogate the “shape” of the distribution. For each sample distribution, the 3^rd^ standardized moment (skewness) and 4^th^ standardized moment (kurtosis) was calculated. The skewness and kurtosis were used to calculate the bimodality coefficient:

$$BC=\frac{{skewness}^{2}+1}{kurtosis}$$

#### Model Implementation

At the start of each simulation, an in silico population of N cells was generated and M kinetic parameter sets constructed using LHCS. In parallel, kinetic parameter sets were submitted with cell populations to conduct the simulations within an ensemble. Many parameter sets were stiff to numerical solving. To prevent stalled simulation of the overall model, populations or single-cells that failed to solve within and allotted time window were aborted. Upon completion each ensemble was checked for a SP response. Upon completion of all of the models, the single-cell SP response distributions were analyzed for each of the passing ensembles in a model and aggregated for comparison.

### Software

Flow cytometry and Flow Sight imaging cytometry data were processed and analyzed using FlowJo for Mac OS X version 10.0.7, Tree Star, Inc. Statistical analyses of experimental data were performed within Graphpad Prism for Mac OS X version 6.0e. Imaging Cytometry images were segmented using ImageJ and the SCIPY platform in python. Cytometry distribution analyses were performed using MATLAB version 2014a (64-bit), MathWorks Inc, in 64-bit Windows 8.1. Side population simulations were implemented in MATLAB version 2014a for Linux and run in parallel on the PACE cluster at Georgia Tech, which consisted of 64 single core 3.8 GHZ AMD processors with over 240 GB of total RAM available (10 GB per node). The following MATLAB File Exchange entries (accessed on 11/5/14) were implemented in MATLAB to analyze or display cytometry or simulation data: smoothhist2d (13352) [85], tight_subplot (27991) [86], suplabel (7772) [87], redblue (25536) [88], progress monitor (32101) [89], and distributionPlot (23661) [90].

## Supporting Information

S1 Fig Flow Cytometry Density Scatter Plots of a Side Population. Hoechst Red and Blue channel emission in flow cytometry results from a representative sample from a SP assay in A549 cells 4-days after passage. Plots are scatter plot densities with each condition normalized to its respective maximum value. Cells were incubated with 10 μM FTC or DMSO vehicle for 30 minutes prior to 90 minutes of 5 μM Hoechst 33342 staining. The SP is visible as the population of cells in the left quadrant gates of the -FTC condition. The quantification of %SP in this plot was calculated to be 20%. B) A representative sample of SP assay results for A549 cells treated with 100 pM TGFβ for 4 days. The quantification of %SP in this plot was calculated to be 0.12%. C) %SP quantification from control (A) and TGFβ-treated (B) samples for 4 replicates. Plotted as mean ± standard error of the mean. Significance was determined with a t-test, p<0.05, and indicated by the asterisk (*).
(EPS)

S2 Fig Multicolor Flow Cytometry Analysis of Surface Marker Staining Representative density scatter plots of staining of A549 cells maintained in culture for 4 days with 0, 1, 10, and 100 pM TGFβ treatment (rows). Columns correspond to the pairwise plots of PE, PE-Texas Red, and APC detection channels with compensated arbitrary fluorescence units. Surface markers were stained with anti-PE-CF594/E-Cadherin (PE-Texas Red Channel), PE/N-Cadherin (PE Channel), and APC/ABCG2 (APC Channel), antibodies and cells counter-stained with SYTOX Blue to exclude dead cells. Density plots correspond to summary of geometric means displayed in Fig 2A & 2B.
(EPS)

S3 Fig Automated Measurement of %SP with Projection Gating. Hoechst staining data in the SP assay, shown as (A) pseudocolored dot density plots and (B) smoothed pseudocolored dot density plots, are transformed into (C) Hoechst Score probability density functions (PDFs). Hoechst Red and Blue Scores transformations of the untreated control cell populations for +FTC (PDF_+FTC_) and -FTC (PDF_-FTC_) conditions are determined by the mean and standard deviation of the +FTC condition. Hoechst Scores are expressed as units of standard deviations from the mean. Density colormap values are normalized to a common maximum frequency across both conditions. SP gates (gray lines) were set at the 1^st^ percentile level using the Hoechst Scores projection gating approach.
(EPS)

S4 Fig TGFβ Treatment Decreases SP Size. A)–FTC plots of TGFβ-treated cells demonstrate decreasing SP size. Flow cytometry density scatter plots Hoechst staining of -FTC conditions from SP assays of a single replicate at Day 4 (Fig 2D) for 0, 1, 10, and 100 pM TGFβ-treated samples. Shown with SP gates set by each sample’s respective +FTC condition. B) The automated projection gating method correlates with manual gating approaches across the multiple conditions of the TGFβ-treatment time course (Fig 2D).
(EPS)

S5 Fig Side and Non-Side Populations Arise from Individual Cells. A) A schematic of the isolation and expansion of low- and high-ABCG2 expressing clonal cell lines from the parent A549 cell line prior to SP analysis. B) Measurement of SP size in cell populations derived from single-cells expanded in culture for 30 days. Projection gating was used to measure %SP in low- and high-ABCG2 expressing clonal cell lines. Shown with line corresponding to mean.
(EPS)

S6 Fig Hoechst Staining Histograms & PDFs of +FTC & -FTC Conditions.Histograms of Hoechst Red (A) and Hoechst Blue (B) staining of +FTC and -FTC conditions for the control sample in S1A Fig, consisting of 51200 cells and 50329 cells for the +FTC and -FTC conditions, respectively. C) Changes in Hoechst Red and Blue Score statistics in -FTC vs +FTC conditions plotted against their associated %SP. (ΔX = X_-FTC_—X_+FTC_; ΔHRS_mean_ = change in Hoechst Red Score mean, ΔHBS_mean_ = change in Hoechst Blue Score mean, ΔHRS_SD_ = change in Hoechst Red Score standard deviation, ΔHBS_SD_ = change in Hoechst Red Score standard deviation, ΔHSC = change in Hoechst Red & Blue Score covariance). Lines of best fit from linear regression are shown along with the corresponding R^2^ values for each Hoechst statistic.
(EPS)

S7 Fig Measurement of SP Response in tBHQ-treated Cells with Imaging Cytometry A) Hoechst Red and Blue Score coordinates for cells selected at random along the diagonal in the–FTC condition of untreated control cells. Numbers correspond to event number ID values. B) Imaging cytometry data channels used in the SP assay. B) The magnitude of the SP response in control and tBHQ-treated samples are reported as the %SP_proj_. Values plotted as the mean ± standard error of the mean of three biological replicates.
(EPS)

S8 Fig Overview of ΔFTC & ΔSP Calculations & Day 4 Plots. A) ΔFTC plots are generated by subtracting the PDF_+FTC_ distribution from the PDF_-FTC_ distribution. The example shown is the formation of the Day 4 0 pM TGFβ (control) condition (green box) from the difference of the average PDF_-FTC_ and PDF_+FTC_ distributions from 3 experimental replicates. Red regions of the ΔFTC plot correspond to regions that have higher density in the -FTC condition while blue regions have density in the +FTC condition. B) The ΔSP plot for a given sample is generated by subtracting the ΔFTC distribution of the control sample (ΔFTC_ctrl_, green box) from the ΔFTC distribution for the sample, (ΔFTC_D4-1pM_, purple box), which gives rise to the ΔSP (ΔSP_D4-1pM_, orange box). Red regions of the ΔSP plot correspond to regions with higher density in the sample condition while blue regions have higher density in the control condition. C) The ΔFTC and ΔSP plots are displayed for the Day 4 samples in the SP time course experiment. The %SP_proj_ is reported for each sample.
(EPS)

S9 Fig Whole Cell & Nuclear Radii Distributions of A549 Cells A) 2D PDF of Flow Sight imaging cytometry measurements of whole cell and nuclear radii using Hoechst stained cells with FTC. B) 2D Distribution of nuclear radius to whole cell radius ratios plotted against whole cell and nuclear radii. C) Sampling of N cells from a 2D PDF of whole cell and nuclear radii (A) reconstructed as a 2D PDF.
(EPS)

S10 Fig ABCG2 and Hoechst Binding Site Distributions A) ABCG2 surface marker staining data from flow-cytometry studies (Fig 2B, n = 3) converted into PDFs of ABCG2 expression in A549 cells. Expression levels were normalized to the mode of the control condition. B) Distribution of Hoechst binding sites available in each cell, estimated from the averaged Hoechst Blue distribution from all of the +FTC conditions, the genomic DNA content of A549 cells, and Hoechst binding site density in genomic DNA.
(EPS)

S11 Fig Schematics of Kinetic Modeling & *In silico* Flow Cytometry A) Hoechst staining dynamics were simulated at the single-cell level with each cell represented by a set of ODEs governed by mass-action kinetics in a well-mixed three-compartment system. The species, compartments, and reactions are depicted. Each cell differs from the rest of the population in terms of volumes, surface areas, transporter properties, and DNA content. Within a given population, all of the cells share a set of kinetic parameters (k) in common. B) At the completion of the kinetic simulations, the total quantity of free and DNA-bound Hoechst dye species are added up within an individual cell. The free and DNA-bound dyes are converted to Hoechst Red and Blue signals according to their spectral properties. C). Excitation and emission spectra for free and DNA-bound Hoechst species displayed alongside Hoechst Red and Blue channels within our cytometer configuration and modeled *in silico*. Also shown is the excitation laser wavelength (355 nm). D) Signals from the Hoechst Blue and Hoechst Red channels are products of emission from both free and DNA-bound Hoechst, though relative efficiency of emission in the two channels is discrepant.
(EPS)

S12 Fig Estimating Hoechst Transport Kinetics Using a Cell Staining Time Course Simulated Hoechst staining for a population of single-cells (left) were used to find a mean staining signal (center) for 5 and 10 μM Hoechst concentrations. The relative kinetics of the two conditions to the 5 μM condition were compared to similarly normalized experimental data (right). Staining took place in the presence of FTC to inhibit any transporter-mediated efflux.
(EPS)

S13 Fig *In silico* Flow Cytometry Results of Subpopulation Type Response. *In silico* flow cytometry SP plots from an ensemble from Model 3, using an experimental derived distribution of transporter expression. A) Schematic representation of the distribution of single-cell SP responses in a Subpopulation Type response. B) Hoechst Scores PDF_+FTC_ and PDF_-FTC_ plots for different transporter distribution samples drawn from 0, 1, 10, and 100 pM TGFβ experimental conditions. Projection gating (gray line) was used to measure the %SP. C) %SP from the ensemble is compared to the means of experimental conditions. D) Differences in PDF_-FTC_ and PDF_+FTC_ distributions from A are shown as ΔFTC for each transporter sample. ΔSP distributions are differences in ΔFTC distributions compared to the 0 pM sample.
(EPS)

S14 Fig *In silico* Flow Cytometry Results of Full Population Type Response. *In silico* flow cytometry SP plots from an ensemble from Model 2, in which cells within the population express variable transporter concentrations. A) Schematic representation of the distribution of single-cell SP responses in a Full Type response. B) Hoechst Scores PDF_+FTC_ and PDF_-FTC_ plots for different transporter samples whose means were drawn from 0, 1, 10, and 100 pM TGFβ experimental conditions. Projection gating (gray line) was used to measure the %SP. C) %SP from the ensemble is compared to the means of experimental conditions. D) Differences in PDF_-FTC_ and PDF_+FTC_ distributions from A are shown as ΔFTC for each transporter sample. ΔSP distributions are differences in ΔFTC distributions compared to the 0 pM sample.
(EPS)

S15 Fig Simulation Outcomes by Transporter Condition. For each of the transporter conditions (A–transporter number distribution (mode i); B–transporter concentration distribution (mode ii); C–experimental transporter distribution (mode iii)), a total of *M* = 10,000 kinetic parameter sets were generated by LHCS and cell staining simulated for the corresponding ensembles. For each of the 10,000 parameter sets, started for each condition, >95% of the sets successfully simulated the corresponding ensemble whereas <5% of the sets had to be aborted while simulating the corresponding ensembles due to failure to meet simulation time-out restrictions (left). Of the successfully simulated parameter sets, >95% failed to produce a SP response as determined by passing the qualitative selection process (right).
(EPS)

S16 Fig Fit of *in silico* SP Responses to Experimental %SP Values For each of the transporter conditions (A–transporter number distribution (mode i); B–transporter concentration distribution (mode ii); C–experimental transporter distribution (mode iii)), the *in silico* %SP responses were compared to the experimental %SP values (Fig 2). For each of the parameter sets having generated ensembles with SP response, the %SP fit RMSE was included in the histogram. RMSE was calculated as a normalized root mean square error using the MATLAB function *goodnessOfFit*, which range from 1, perfect fit, to negative infinity with progressively worse fit to experimental data.
(EPS)

S17 Fig Sampled Parameters Compared to SP Response Parameters For each of the transporter conditions (A–transporter number distribution (mode i); B–transporter concentration distribution (mode ii); C–experimental transporter distribution (mode iii)), histograms of the kinetic parameter sets were sampled using LHCS of Log_10_-uniform distributions from the lower to the upper limit of the sampled range (blue) are shown. Parameter sets with corresponding ensembles generating SP responses (red) are a subset of the sampled distribution.
(EPS)

S18 Fig Distributions of -ΔH_proj_ for Subpopulation and Full Response Types. The -ΔH_proj_ of cells in each transporter sample population of an ensemble is displayed in a histogram for a Subpopulation Type (A) and a Full Type Response (B). The standard skewness (S), excess standard kurtosis (k), and bimodality coefficient (B) is listed in the upper right-hand corner for each distribution.
(EPS)

S19 Fig Single-Cell Analysis of Hoechst Projections & -ΔH_proj_. *In silico* single-cell Hoechst Projection plots for the 0 pM TGFβ sample for a Subpopulation Type and a Full Type response. A) Hoechst Score Projections in the uninhibited condition (H_proj_-FTC) are plotted against Hoechst Score Projections in the inhibited condition (H_proj_+FTC). B) The change in Hoechst Score Projection (-ΔH_proj_) is plotted as a function of number of transporters per cell. C) -ΔH_proj_ is plotted as a function of Hoechst Score Projection in the inhibited condition (H_proj_+FTC). D) -ΔH_proj_ is plotted as a function of Hoechst Score Projection in the uninhibited condition (H_proj_-FTC).
(EPS)

S20 Fig SP Response Distribution Landscape. Distributions of SP response (-ΔH_proj_) in individual cells within the populations of ensembles meeting SP selection criteria were characterized by standardized skewness and bimodality. Simply put, skewness measures the degree of asymmetry of a distribution around the mean with a value of 0 corresponding to symmetry and positive values corresponding to distributions with larger ranges in the distribution above the mean. Likewise, negative skew values correspond to distributions with a larger range in the distribution below the mean than above it. The bimodality coefficient is calculated from the standardized skewness and standardized kurtosis. It has a range from 0 to 1, in which a value of 0 reflects a distribution with a single value while 1 corresponds to a distribution with exactly two values. Distributions with two fairly distinct modes score closer to 1 while distributions with a singular mode with a higher frequency score closer to 0. The mappings of a wide variety of example distributions are depicted along with representations of the range single-cell SP responses in an example cell population. Lower case letters correspond to positioning on the Response Distribution Map.
(EPS)
